# Microbiological profile and risk factors for in-hospital mortality of infective endocarditis in tertiary care hospitals of south Vietnam

**DOI:** 10.1371/journal.pone.0189421

**Published:** 2017-12-14

**Authors:** Hoang M. Tran, Vien T. Truong, Tam M. N. Ngo, Quoc P. V. Bui, Hoang C. Nguyen, Trung T. Q. Le, Wojciech Mazur, Eugene Chung, John M. Cafardi, Khanh P. N. Pham, Hoang H. N. Duong, Thach Nguyen, Vu T. Nguyen, Vinh N. Pham

**Affiliations:** 1 Pham Ngoc Thach university of medicine, Ho Chi Minh city, Viet Nam; 2 The Christ Hospital, Cincinnati, Ohio, United States of America; 3 St. Mary Medical Center, Hobart, Indiana, United States of America; University of California San Francisco, UNITED STATES

## Abstract

**Objectives:**

We aimed to evaluate the microbiological characteristics and risk factors for mortality of infective endocarditis in two tertiary hospitals in Ho Chi Minh City, south Vietnam.

**Materials and methods:**

A retrospective study of 189 patients (120 men, 69 women; mean age 38 ± 18 years) with the diagnosis of probable or definite infective endocarditis (IE) according to the modified Duke Criteria admitted to The Heart Institute or Tam Duc Hospital between January 2005 and December 2014.

**Results:**

IE was related to a native valve in 165 patients (87.3%), and prosthetic valve in 24 (12.7%). Of the 189 patients in our series, the culture positive rate was 70.4%. The most common isolated pathogens were *Streptococci* (75.2%), *Staphylococci* (9.8%) followed by gram negative organism (4.5%). The sensitivity rate of *Streptococci* to ampicillin, ceftriaxone or vancomycin was 100%. The rate of methicillin resistant *Staphylococcus aureus* was 40%. There was a decrease in penicillin sensitivity for *Streptococci* over three eras: 2005–2007 (100%), 2008–2010 (94%) and 2010–2014 (84%). The in-hospital mortality rate was 6.9%. Logistic regression analysis found prosthetic valve and NYHA grade 3 or 4 heart failure and vegetation size of more than 15 mm as strong predictors of in-hospital mortality.

**Conclusion:**

Streptococcal species were the major pathogen of IE in the recent years with low rates of antimicrobial resistance. Prosthetic valve involvement, moderate or severe heart failure and vegetation size of more than 15 mm were independent predictors for in-hospital mortality in IE.

## Introduction

Despite major advances in therapeutic and diagnostic options, mortality and morbidity associated with infective endocarditis (IE) has not decreased significantly in the past four decades [1]. This may be related to factors such as increased frequency of age-related valvular degeneration, prosthetic-valve surgery, and hospital-related infections that change the microbial flora and antibiotic susceptibility [1–4].

Classically, *Streptococci* have been the main causative microorganisms of IE. However, recent studies have shown a significant increase in frequency of *Staphylococcus aureus*, up to 30% of cases [1]. As recent IE treatment recommendations are significantly based on non-randomized studies and expert opinion [5, 6], empiric antibiotic therapy is usually applied based on local microbiological characteristics.

For this reason, it is essential to periodically update information about regional IE pathogen characteristics and antibiotic susceptibility profile. The aim of this study was to evaluate the microbiological characteristics as well as factors associated with increased in-hospital mortality in patients hospitalized for infective endocarditis at two tertiary care hospitals in South Vietnam.

## Materials and methods

### Study design

This study was performed at Heart Institute and Tam Duc Hospital of Cardiology, which are tertiary care referral hospitals located in Ho Chi Minh City, South Vietnam, between 01/01/2005 to 12/31/2014. The hospital charts of patients admitted with a diagnosis of IE according to the modified Duke criteria [6, 7] were retrospectively reviewed. Patients with lack of microbiological results were excluded. A total of 189 consecutive patients with diagnosis of definite or probable IE were eligible for inclusion with 17 patients excluded from study because of lack of microbiologic data. This study was approved by the institutional review board (IRB) of Pham Ngoc Thach university of medicine as well as IRB of Heart Institute and Tam Duc Hospital of Cardiology. Informed consent was waived because of the retrospective nature of the study.

The following variables were collected for each patient:

Clinical background: age, sex, factors predisposing to infective endocarditis (valvular heart diseases, congenital heart diseases, prosthetic valve, pacemaker implantation, history of injection drug use), history of cardiac surgery, medical comorbidities (including diabetes, hypertension, chronic kidney disease and ischemic heart disease), clinical signs and symptoms and the presence of systemic embolic disease.

Abnormal l*ab*oratory data: acute renal failure (increase of serum creatinine > 26.5 μmol/l within 48 hours), white blood cell count (WBC) > 11.000 cells/L, C reactive protein (CRP) concentration > 100 mg/l.

Findings on ECG: any rhythm other than sinus tachycardia (heart rate > 100 beats/min).

Findings on echocardiography: location of visible vegetation, vegetation size, vegetation number, valve type, impaired left ventricular function (ejection fraction < 40%), congenital heart diseases, intracardiac complications of infective endocarditis.

### Statistical analysis

Continuous variables are expressed as mean **±** standard deviation (SD) for normal distributions and median + interquartile range for non-normal distributions. Categorical variables were represented as frequencies and percentages. For the evaluation of qualitative variables, we used the Chi-Square test. To test for significant differences between continuous variables in two groups, independent sample t-tests were performed. The patient variables that were analyzed in the univariate analysis included age, gender, valve type, heart failure, systemic emboli, conduction abnormalities, congenital heart diseases, WBC, CRP, acute renal failure, vegetation site, vegetation size, ejection fraction, intracardiac complications, positive blood culture, *Staphylococcus aureus* infection. Logistic regression analysis was performed to identify independent prognostic factors for death. [8]. Statistical analysis was performed using the SPSS 22 software program (SPSS Inc., Chicago, IL, USA). A p value of < 0.05 was considered statistically significant.

## Results

### Baseline characteristics

During this 10-year period, a total of 189 consecutive patients with diagnosis of definite or probable IE were identified (S1 File). Baseline characteristics, predisposing conditions, clinical findings on admission for the 189 IE cases are shown in Table 1. The mean age of patients was 37.6 ± 18.0 years with 120 men (63.5%) and 69 women (36.5%).

**Table 1:** Characteristics of study sample.

Features	n (%)	Features	n (%)
Clinical background		Symptoms and signs	
Valvular heart diseases	125 (66.1)	Fever	168 (88.9)
Congenital heart diseases	36 (19.1)	Fever duration (days) before admission	26.69 ± 24.64
Prosthetic valve	24 (12.7)	Rigors	15 (7.9)
Pacemakers implantation	4 (2.1)	Dyspnea	66 (34.9)
History of cardiac surgery	33 (17.5)	Anorexia	10 (5.3)
History of infective endocarditis	12 (6.3)	Weight loss	12 (6.3)
Intravenous drug user	2 (1.1)	Fatigue	46 (24.3)
Previous antibiotic usage	46 (24.3)	Cardiac murmur	115 (60.8)
Hypertension	21 (11.1)	Hepatomegaly	21 (11.1)
Diabetes	2 (1.1)	Splenomegaly	6 (3.2)
Chronic kidney disease	2 (1.1)	Roth spot	6 (3.2)
Ischemic heart disease	3 (1.6)	Osler’s node	1 (0.5)
		Skin rash	4 (2.1)
		Embolisms	19 (10.1)
		Cardiac conduction disorder	7 (3.7)

Categorical variables are presented as n (%)

33 patients (17.5%) had a history of cardiac surgery while 2 patients (1.1%) had a history of intravenous drug abuse. Only 2 patients (1.1%) had diabetes while none had end-stage renal disease. Predisposing valvular heart disease was found in 125 (66.1%) and congenital heart diseases in 36 (19.1%) of the patients. Prosthetic cardiac valves were present in 24 (12.7%) and 4 (2.1%) patients experienced pacemaker lead IE (Table 1).

In our study, vegetation was observed in 172 cases (91%), of whom 14 (7.4%) were found to have large vegetation (>15 mm). Affected valves were mitral valve in 78 (41.3%), aortic valve in 38 (20.1%), mitral and aortic valves in 11 (5.8%), tricuspid valve in 8 (4.2%) patients, pulmonary valve in 6 (3.2%) patients and right ventricular wall in 15 (7.9%) patients. Other vegetation sites were observed in 16 patients (8.5%) (Table 2).

**Table 2:** Echocardiographic findings of the study sample.

**Vegetation number**	**n (%)**	**Vegetation site**	**n (%)**
0	17 (9.0)	Mitral	78 (41.3)
1	87 (46.0)	Aortic	38 (20.1)
2	46 (24.3)	Mitral and aortic	11 (5.8)
≥ 3	39 (20.7)	Tricuspid	8 (4.2)
**Vegetation size**	**n (%)**	Pulmonary	6 (3.2)
≤ 10mm	116 (61.4)	Tricuspid and pulmonary valve	2 (1.1)
10-15mm	59 (31.2)	Left and right sides	5 (2.6)
>15mm	14 (7.4)	Right ventricular wall	15 (7.9)
**Intracardiac complications**	**n (%)**	Pulmonary arterial wall	6 (3.2)
Valve leaflet perforation	41 (62.1)	Left ventricular wall	1 (0.5)
Chordae tendinae rupture	20 (30.3)	Pacemaker wire	2 (1.1)
Paravalvular abscess	19 (28.8)	No vegetation	17 (9.0)
Prosthetic valve dehiscence	3 (4.5)		

Categorical variables are presented as n (%)

### Microbiological data

Blood cultures were performed in all patients, with a positive rate of 70.4% (133 patients). There was a significant difference in positive culture rate between patients with or without prior antibiotic use before admission (50% and 76.9%, respectively; p = 0.001). *Streptococci* remained the most common causative agent of IE (75.2%), with Staphylococcal species identified in 13 patients (9.8%). Eleven of these 13 patients had *Staphylococcus aureus* (Table 3).

**Table 3:** Causative microorganisms of 133 cases of culture positive infective endocarditis.

Pathogens	Cases (n)	(%)
*Streptococci*	100	75.2
Viridans group *Streptococci*	91	68.4
Other *Streptococci*	9	6.8
*Staphylococci*	13	9.8
*Staphylococcus aureus*	11	
*Staphylococcus epidermidis*	2	
*Enterococcus faecalis*	5	3.8
Gram negative bacteria	6	4.5
*Pseudomonase aeruginosa*	2	
*Stenotrophomonas maltophilia*	1	
*Burkholderia cepacia*	2	
*Acinetobacter baumani*	1	
Anerobic bacteria	2	1.5
*Gemella hemolysans*	1	
*Gemella morbillorum*	1	
Other agents	4	3.0
*Chryseobacterium indologenes*	1	
*Granulicatella adiacens*	1	
*Haemophilus influenzae*	1	
*Weeksella virosa*	1	
*Candida* spp.	3	2.2

Categorical variables are presented as n (%)

Over the three time periods examined, no significant changes were observed regarding infectious endocarditis microbiology (p = 0.059) apart from an increased frequency of Staphylococcal infection in the last period (18.2% versus 4.7% and 2.9%) (Table 4). The data showed no statistically significant differences regarding the causative pathogens rate between the groups of patients having early and late infective endocarditis (p = 0.1). However, there was a higher rate of Staphylococcal infection in patients having prosthetic valve compared to native valve (16.7% versus 9.1%, P = 0.01) (Table 4). Methicillin-resistant *staphylococcus aureus* (MRSA) accounted for 40% of *Staphylococcus aureus*. Streptococcal species were sensitive to ceftriaxone, ampicillin and vancomycin in 100% of cases; they were sensitive to penicillin 92.7% of the cases.

**Table 4:** Causative microorganisms over the three time periods.

Causative microorganisms	Valve nature		Time to IE		Vegetation site			Period		
	Native valve IE	Prosthetic valve	Early IE	Late IE	Left side IE	Right side IE	Both sides	2005–2007	2008–2010	2011–2014
*Streptococci*	94	6	2	7	67	22	2	31	34	35
	(77.7)	(50)	(18.2)	(77.8)	(82.7)	(64.7)	(40)	(88.6)	(79.1)	(63.6)
*Staphylococci*	11	2	4	1	4	6	1	1	2	10
	(9.1)	(16.7)	(36.4)	(11.1)	(4.9)	(17.6)	(20)	(2.9)	(4.7)	(18.2)
Gram negative bacilli	4	2	2	0	4	1	0	0	4	2
	(3.3)	(16.7)	(18.2)	(0)	(4.9)	(2.9)	(0)	(0)	(9.3)	(3.6)
Other bacteria	11	0	1	0	4	4	2	2	2	7
	(9.1)	(0)	(9.0)	(0)	(4.9)	(11.8)	(40)	(5.7)	(4.7)	(12.7)
Candida spp.	1	2	2	1	2	1	0	1	1	1
	(0.8)	(16.7)	(18.2)	(11.1)	(2.5)	(2.9)	(0)	(2.9)	(2.3)	(1.8)
Total	121	12	11	9	81	34	5	35	43	55
	(100)	(100)	(100)	(100)	(100)	(100)	(100)	(100)	(100)	(100)

### In-hospital mortality and predictive factors

Thirteen of 189 patients died (6.9%) during their hospital stay. In the univariate analysis, risk factors that increased mortality were: prosthetic valve involvement, severe heart failure (NYHA classification 3 or 4), systemic emboli complication, conduction abnormalities, acute renal failure, vegetation size > 15 mm, intracardiac complications, undefined microorganism by blood culture (Table 5). While, prosthetic valve involvement (OR = 34.97, P = 0.006) and severe heart failure (NYHA 3, 4) (OR = 21.91, P = 0.01), vegetation size > 15 mm (OR = 23.29, p = 0.029) were important independent risk factors for mortality in adjusted analysis.

**Table 5:** Factors associated with in-hospital mortality, unadjusted.

Factor	Category	Number	Deaths (%)	OR	95% CI	P value
Age (years)	<55	146	10 (6.8)	1	0.27–3.89	0.977
	≥55	43	3 (7.0)	1.02		
Gender	Female	69	2 (2.9)	1	0.73–15.72	0.120
	Male	120	11 (9.2)	3.38		
Valve type	Native valve	165	7 (4.2)	1	2.28–24.84	0.001
	Prosthetic valve	24	6 (25)	7.52		
Heart failure grade	NYHA ≤ 2	142	3 (2.1)	1	3.28–47.83	<0.0001
	NYHA 3, 4	47	10 (21.3)	12.52		
Systemic emboli	No	175	9 (5.1)	1	1.93–28.16	0.003
	Yes	14	4 (28.6)	7.38		
Conduction abnormalities	No	182	10 (5.5)	1	2.54–65.65	0.002
	Yes	7	3 (42.9)	12.9		
Congenital heart disease	No	153	11 (7.2)	1	0.16–3.59	0.728
	Yes	36	2 (5.6)	0.76		
Elevated leucocyte count	No	86	4 (4.7)	1	0.58–6.61	0.276
	Yes	103	9 (8.7)	1.96		
Elevated CRP	No	141	9 (6.4)	1	0.41–4.77	0.594
	Yes	46	4 (8.7)	1.4		
Acute renal failure	No	159	8 (5.0)	1	1.14–12.47	0.029
	Yes	30	5 (16.7)	3.78		
Vegetation size	≤ 15 mm	175	9 (5.1)	1	1.93–28.16	0.003
	>15 mm	14	4 (28.6)	7.38		
Vegetation site	Pure left IE	128	8 (6.2)	1	0.17–3.99	0.796
	Pure right IE	39	2 (5.1)	0.81		
Ejection fraction	≥ 40%	182	12 (6.6)	1	0.83–240.77	0.067
	< 40%	2	1 (50)	14.17		
Intracardiac complications	No	122	3 (2.5)	1	1.84–26.27	0.004
	Yes	67	10 (14.9)	6.96		
Blood culture	Positive	133	5 (3.8)	1	1.33–13.69	0.015
	Negative	56	8 (14.3)	4.27		
Staphylococcus aureus	No	178	12 (6.7)	1	0.16–11.73	0.766
	Yes	11	1 (9.1)	1.38		

Categorical variables are presented as n (%); OR: odds ratio; CI: confidence interval

## Discussion

The mean age of patients in our study was 37.6 ± 18.0 years. Published studies from developing countries also reported that patients with IE were mostly young [9–11]. Letaief *et al* reported on the epidemiology of infective endocarditis in Tunisia, showing a mean age of 32.4 ± 16.8 years [11]. This is contrast to data from developed countries which consistently report an older population with IE (median age 57.9 (IQR 43.2–71.8) years) [2]. This may be explained by high prevalence of rheumatic heart disease in Vietnam, whereas degenerative valve disease was the most common form of valvular disease in developed countries [2]. A Turkish study showed that the main factor contributing to younger patient age in IE could be the higher rate of rheumatic heart disease [12]. Mirabel *et al* studied infective endocarditis in the Lao PDR found patients with IE were mostly younger, and the most predisposing condition was rheumatic heart disease [9].

Echocardiography plays a key role in the diagnosis of IE. It is very useful to identify vegetations associated with IE as well as the assessment of complications of the disease. TEE is superior to TTE for detection of valvular vegetation as well as cardiac complications such as abscess, valvular leaflet perforation, chordae tendinae rupture and pseudoaneurysm [13, 14]. In our study, the vegetation rate was found up to 91%. Regarding to vegetation site, mitral valve was the most commonly affected valve, followed by the aortic valve, which is similar to the reports from the previous study [15].

The culture positive rate was 70.4%. This rate is higher than many studies from other developing countries [9, 10] but remains lower than those from developed countries [16, 17]. The high negative blood culture in our study can be explained by patients’ self-medication with antibiotics, which is common in Vietnam [18–20]. In addition, detection of fastidious organisms is challenging with the use of conventional blood culture techniques, as isolation of these organisms requires special media or cell culture conditions. In addition, we lacked other techniques used to diagnose the pathogens of culture negative IE such as serological analysis for *Coxiella burnetii* and *Bartonella* species, polymerized chain reaction assays for *T*. *whipplei* or *Bartonella* and ribosomal RNA PCR assays on valvular specimens [21].

Under these conditions, our findings showed the most frequent causative microorganism to be *Streptococci* (75.2%), *Staphylococci* (9.8%), and gram negative bacilli (4.5%). With regard to the microbiology of IE over the three time periods, no significant changes were observed regarding infectious endocarditis microbiology, although there has been an increasing frequency of Staphylococcal species. The high prevalence of Streptococcal species in our study differs from the high rates of Staphylococcal infection noted in recent studies from the developed world [2, 16, 17]. There are several reasons for this discrepancy. First, the patients in this study had a lower mean age and a higher prevalence of rheumatic heart disease, however they had lower rates of persistent bacteremia, hemodialysis, diabetes, and intravascular devices, which are key risk factors associated with IE due to *Staphylococcus aureus* [22]. Second, the oral health status of the Vietnamese population is sub-optimal [23, 24], which is a key predisposing cause of IE. That could also explain why *Streptococci*, especially viridans group *Streptococci* was the most common observed pathogen. Finally, prosthetic valve prevalence, intravenous line-related IE and injection drug abuse was low compared with other studies.

Antimicrobial resistance was not a major problem among the microorganisms isolated from community-acquired endocarditis in our study. All *Streptococci* were sensitive to ampicillin, ceftriaxone, and vancomycin, while there was a mildly reduced susceptibility to penicillin, consistent with the worldwide increase in penicillin resistant viridans group *Streptococci*. For *Staphylococci*, all isolates were susceptible to vancomycin and teicoplanin, but there was high rate of methicillin resistant *Staphylococcus aureus* (40%). We suspect that the higher rate of methicillin resistance in our study reflects in part the widespread consumption of antimicrobials in the community in Vietnam, although this is consistent with the increased rate of methicillin resistance observed worldwide.

Our study shows in-hospital mortality rate of 6.9%, which is lower than previous reports [2, 25, 26]. The characteristic of our study sample which includes mostly younger patients affected by Streptococcal infections with lower rate of comorbidities likely explains the low observed mortality. Indeed, viridans Streptococcal IE has been documented as having a good prognosis versus other pathogens [2], as well as younger age [2, 26]. In our study, several factors were associated with in-hospital mortality, including moderate or severe heart failure, prosthetic valve involvement, systemic emboli complication, conduction abnormalities, vegetation length, intracardiac complication and negative blood culture. The strong predictors were prosthetic valve involvement, moderate or severe heart failure, vegetation size > 15 mm. Congestive heart failure has been repeatedly reported as the common cause of death in infective endocarditis [27–29]. Prosthetic valve involvement and vegetation length > 15 mm were also associated with mortality in previous studies [10, 27]. Published studies have found other predictors of mortality. Hasbun et al found abnormal mental status, moderate to severe heart failure, comorbidity, staphylococcal infection, and medical therapy without valve surgery were independent predictors for mortality at 6 months [28]. In addition, Thuny et al showed that clinical indicies such as age, female sex, creatinine serum > 2mg/l, moderate or severe congestive heart failure, staphylococcal infection and vegetation length > 15 mm were strong predictor of 1-year mortality [27].

In conclusion, in this Vietnamese population in recent years, streptococcal species were the major pathogen associated with IE with low rates of observed antimicrobial resistance. Prosthetic valve involvement, moderate or severe heart failure and vegetation size > 15 mm were the most important independent predictors for in-hospital mortality in IE.

## Study limitations

The main limitation of this study is that the data were collected from only 2 tertiary care hospitals. Other than the relatively small sample size, there may also be a referral bias, and we cannot conclude patients with IE in the broader communities of Vietnam. The relatively low frequency of injection drug use may be due to reporting bias. We also cannot exclude selection bias, that is that the most severe cases of IE died before diagnosis and transport to our hospitals. The risk factors for in-hospital mortaliy may not be reliable due to the low mortality rate, which leads to wide 95% confidence interval. Finally, the retrospective study design does not allow rigorous long term follow up of patients.

## Supporting information

S1 File Raw dataset of the study. (XLS)
